# THE USABILITY AND ACCEPTABILITY OF THE AUGMENTED REALITY HOME ASSESSMENT TOOL (ARHAT)

**DOI:** 10.1093/geroni/igad104.3668

**Published:** 2023-12-21

**Authors:** Beth Fields, Zachary Skrove, Ross Tredinnick, Bryce Sprecher, Jenny Lee, Rachael Shields, Kevin Ponto, Jung-hye Shin

**Affiliations:** University of Wisconsin-Madison, Madison, Wisconsin, United States; University of Wisconsin-Madison, Madison, Wisconsin, United States; University of Wisconsin-Madison, Madison, Wisconsin, United States; University of Wisconsin-Madison, Madison, Wisconsin, United States; University of Wisconsin-Madison, Madison, Wisconsin, United States; University of Wisconsin-Madison, Madison, Wisconsin, United States; University of Wisconsin-Madison, Madison, Wisconsin, United States; University of Wisconsin-Madison, Madison, Wisconsin, United States

## Abstract

Gaining access to reliable home assessments for aging in place can be challenging and expensive for urban residents and particularly those living in rural areas. Evidence has shown that there is a substantial increase in the number of older adults nationally and globally, making the availability of home assessments scarce. As individuals continue to age and become more susceptible to disabilities it is important that they have a home that allows them to age in place and is compliant with housing standards. Technology has the potential to make home assessments more accessible to older adults who want to age in place. The aim of this study was to determine the usability and acceptability of a mobile-based app, the Augmented Reality Home Assessment Tool (ARHAT), across different groups interested in home assessment (occupational therapists (OTs), older adults/family caregivers, and housing professionals) (N=16). Participants were mailed an iPhone with the ARHAT installed. After participants reviewed the ARHAT, they were emailed an electronic survey with closed and open-ended demographic, usability and acceptability questions. The majority of participants were White (94%), female (75%), from the Midwest (63%), and had a Bachelor’s degree or higher (81%). Participants reported that the ARHAT is easy to use and acceptable for measuring features of the home environment and can support aging in place. Future research should test the impact of the ARHAT on improving health service utilization (e.g., delaying institutionalization, reducing emergency department visits) rates among older adults.

